# Glycine receptor antibodies and coeliac disease-related neurological dysfunction

**DOI:** 10.1186/s40673-021-00135-3

**Published:** 2021-05-03

**Authors:** Lewis Kass-Iliyya, Ptolemaios G. Sarrigiannis, David S. Sanders, Marios Hadjivassiliou

**Affiliations:** Academic Department of Neurosciences, Sheffield Teaching Hospitals NHS Foundation Trust, Sheffield, UK

**Keywords:** Gluten sensitivity, Coeliac disease, Glycine receptor antibodies, Brain hyperexcitability, ataxia

## Abstract

Gluten sensitivity can manifest with a spectrum of neurological dysfunction including ataxia, encephalopathy and neuropathy with or without associated coeliac disease (CD). Gluten sensitivity can also present with central nervous system (CNS) hyperexcitability and cortical myoclonus which is often accompanied with refractory CD. CNS hyperexcitability can also be associated with Glutamic Acid Decarboxylase (GAD) antibodies or much less commonly with Glycine Receptor Antibodies (GlyR-Abs) but the direct pathogenic roles of these antibodies remain debatable. We have previously reported a link between gluten sensitivity and anti-GAD associated ataxia which improves with the adoption of gluten-free diet. It is unclear if a similar link exists between gluten driven CNS hyperexcitability and the presence of GlyR-Abs. We report two cases of CD presenting with CNS hyperexcitability and associated GlyR-Abs. Apart from ataxia and cortical myoclonus, one patient had refractory CD and died from enteropathy-associated T-cell lymphoma. The other patient not only improved with strict gluten-free diet but also showed serological elimination of circulating GlyR-Abs. We conclude that there is an interaction between gluten sensitivity and GlyR-Abs-associated CNS hyperexcitability and in such patients gluten-free diet is an important therapeutic intervention. The elimination of GlyR-Abs by the adoption of gluten free diet suggests that these antibodies may represent an epiphenomenon rather than being directly implicated in the pathogenesis.

## Introduction

Gluten sensitivity represents a spectrum of disorders triggered by the ingestion of gluten [1]. The diagnosis of gluten sensitivity relies on serological evidence of antibodies linked to gluten sensitivity (one or more of antigliadin, TG2, endomysium-EMA, TG6 antibodies). Some of these antibodies (TG2 and EMA) are specific to the presence of enteropathy (coeliac disease-CD). For those gluten sensitivity cases where there is no evidence of enteropathy, and the manifestations are often extraintestinal (such as neurological), antigliadin antibodies (AGA) and TG6 antibodies can be the only markers. Neurological manifestations can therefore be present in the absence of enteropathy [1].

Various neurological manifestations have been described in the context of gluten sensitivity the commonest of which are ataxia, neuropathy and encephalopathy [1]. Other less common presentations include central nervous system (CNS) hyperexcitability conditions such as cortical myoclonus with ataxia and stiff person syndrome [2, 3]. Indeed, more than half of the patients presenting with neurological illness due to gluten sensitivity do not have CD [4]. Frequently the adoption of a strict gluten-free diet results in elimination of the antibodies related to gluten sensitivity as well as clinical improvement [5].

Stiff person Syndrome (SPS) is a rare autoimmune neurological disorder characterised by axial muscle stiffness and spasms, often accompanied by neuropsychiatric symptoms. SPS is usually associated with glutamic acid decarboxylase (GAD) antibodies found in 70% of cases [6]. Variants of SPS include paraneoplastic SPS (typically associated with amphiphysin antibodies) and progressive encephalomyelitis with rigidity and sometimes myoclonus (PERM) which, in addition to the classic symptoms, manifest with brainstem signs. PERM is reported to be associated with glycine receptor antibodies (GlyR-Abs) [7]. The pathogenic role of both GAD antibodies and GlyR-Abs remains debated. Both types of antibodies have been found to occur in other autoimmune conditions with varied phenotypes including ataxia, limbic encephalitis and epilepsy [8–10].

We have previously reported a significant overlap between gluten sensitivity and anti-GAD associated diseases. In our cohort of gluten sensitive patients we found anti-GAD antibodies to be present in a high proportion of patients who also displayed features of stiff person syndrome or ataxia [3, 11]. Furthermore, strict gluten-free diet resulted in serological reduction of the anti-GAD titre and in some cases reversal from anti-GAD positive to anti-GAD negative which corresponded to clinical improvement [3]. This raised the question whether gluten sensitivity and anti-GAD related neurological disease are part of the same disease spectrum and that gluten may be the driver of the autoimmunity.

Brain hyperexcitability is also seen in patients with gluten sensitivity with and without CD although patients with persistent cortical myoclonus tend to have refractory CD [2]. It is unclear if there is a link between gluten related brain hyperexcitability and positive GlyR-Abs. We describe here 2 patients with CD and GlyR-Abs with clinical and electrophysiological features of CNS hyperexcitability.

## Case reports

### Patient 1

This was a 72-year-old man with a past medical history of hypertension and immune mediated hypothyroidism. He first presented to secondary care with a history of weight loss. His biochemistry profile revealed iron deficiency anaemia, low vitamin D, low folate and normal vitamin B12. CD was suspected and serological testing showed positive tissue transglutaminase antibodies (TTG), gliadin antibodies and endomysium antibodies (EMA). Gastroscopy and duodenal biopsy confirmed villous atrophy, crypt hyperplasia and increased intra-epithelial lymphocytes consistent with CD. He commenced gluten-free diet. Six months later, he presented with worsening mobility and further weight loss and at that point he was noted to have marked ataxia and right facial myoclonus that was speech sensitive. He admitted that he was not adhering to a strict gluten-free diet.

**Fig. 1:**
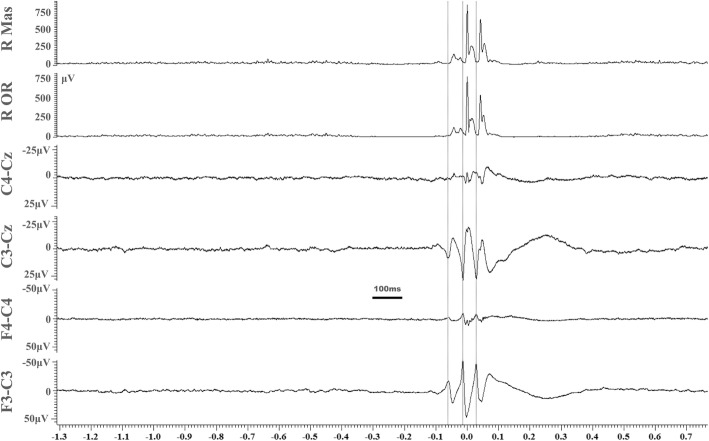
Results from the technique of jerk-locked averaging after triggering events from the short duration EMG discharges captured from the right hemiface. The electrophysiological findings show clear evidence of cortically-driven myoclonus from the right lower hemiface. The three vertical cursors mark the onset of the averaged and rectified EMG discharges from the right hemiface. There is a time lag of 9 ms between the onset of the EMG discharges and the biphasic spikes appearing in the contralateral frontocentral and central EEG electrodes. In total 103 sweeps were averaged. R Mass: right masseter, R OR: right orbicularis oris

Neurophysiological assessment was undertaken with a Natus Quantum amplifier (Optima Medical Ltd., Guildford Surry, UK) at a sampling rate of 2048 Hz. The recording included a multichannel electroencephalography (EEG) and surface EMG polygraphy (analogue bandwidth 0.01–680 Hz). Data were exported for quantitative EEG/EMG analysis in Spike 2 (version 8.12) software (CED Ltd., Cambridge, UK). Somatosensory evoked potential (SEP) recordings were also performed and the possibility of C-reflexes (cortical reflexes looking for cortical reflex myoclonus) was assessed with surface EMG electrodes from arms and legs based on previously published methods [12, 13]. The recording showed clear evidence on jerk-locked averaging of cortically driven myoclonus (Fig. 1). There was no abnormality in the SEP studies, specifically no evidence of “Giant” SEPs or any suggestion of C-reflexes.

Further serological testing revealed positive GlyR-Abs (using a cell based assay) and negative GAD antibodies. Paraneoplastic antibodies were also negative. MRI spectroscopy was markedly abnormal with N-Acetyl-Aspartate to Creatinine (NAA/Cr) ratio of 0.67 from the superior vermis and 0.61 from the cerebellar hemisphere (normal value > 1). Reduced NAA/Cr is indicative of cerebellar neuronal dysfunction. PET-CT imaging revealed no malignancy. His neurological symptoms continued to worsen. Further immunohistochemistry on the original duodenal biopsy showed clonal changes supporting a diagnosis of refractory CD type 2 but without any evidence of enteropathy associated lymphoma. The patient was started on budesonide and mycophenolate. Levetiracetam was tried for the myoclonus. He continued to deteriorate clinically primarily due to worsening ataxia and eventually required PEG feeding as well as becoming bed bound. A repeat duodenal biopsy showed enteropathy-associated T-cell lymphoma (EATL). At that point he was deemed unfit for chemotherapy and passed away in hospital shortly after from pneumonia. This was 1 year after his initial presentation.

### Patient 2

This was a 20-year old man who presented with 8-year history of progressive painful leg spasms and extreme fatigue. He experienced difficulty with running which he used to enjoy. He felt that his muscles were becoming increasingly stiff every time he went for a run. His family also noted cognitive slowing which interfered with his academic performance. Initial investigations by his General Practitioner showed him to be iron deficient. As a result, screening for CD revealed an extremely high level of TTG antibodies, EMA and gliadin antibodies. Duodenal biopsy confirmed CD and he started a gluten-free diet. He was referred to the Gluten sensitivity/neurology clinic in Sheffield UK. Examination revealed ataxic gait, increased tone and stiffness in both lower limbs with hyper-reflexia. He had exaggerated startle response. Imaging of the neural axis did not show any abnormality apart from reduced NAA/Cr ratio of 0.81 from the vermis. The ratio from the right cerebellar hemisphere was 0.9 (normal > 1).

EEG/EMG polygraphy undertaken as described in the previous patient did not show evidence of cortically driven myoclonus from the right thigh on cross correlation analysis. There was no abnormality in the SEP study. Exaggerated startle response was noted following presentation of an unanticipated auditory stimuli. Blink reflex excitability studies (implemented as a semiquantitative assessment of brainstem excitability) while using a short inter-stimulus interval between a conditioning and a test stimulus after 160 ms were performed. Single square pulse electrical stimulation of the supraorbital nerve was delivered at 20–25 mA and a 0.2 s pulse width. The blink reflex study was noted to be abnormal with persistence of a well formed R2 component recorded following the test stimulus. This electrophysiological assessment is used as a semiquiantitative assessment of brainstem excitability [14].

GlyR-Abs were positive and anti-GAD negative. After adopting a strict gluten-free diet he noticed gradual improvement in his symptoms. He was now able to run again and resume his academic career. After 2 years on strict gluten-free diet and normalisation of the gluten sensitivity-related antibodies his glycine receptor antibodies became negative. Repeat electrophysiology showed normalisation of the blink reflex study. Repeat brain image showed improvement of the NAA/Cr ratio from the vermis (from 0.81 to 0.88).

## Discussion

We have previously identified a link between gluten sensitivity and anti-GAD associated SPS as well as anti-GAD ataxia [3, 11]. Here we report a link between coeliac disease-related CNS hyperexcitability and GlyR-Abs. Both of our patients had clinical and neurophysiological features of brain hyperexcitability. The first patient went on to develop enteropathy associated lymphoma (related to refractory CD) and died and as such we have no long term follow up information. The second patient responded well to gluten-free diet, not only demonstrating clinical improvement with elimination of the CD related antibodies but also with evidence of elimination of GlyR-Abs. We routinely screen all our patients with suspected autoimmune ataxia for the presence of GlyR-Abs and in our cohort of more than100 patients tested we have only found GlyR-Abs to be positive in these two cases. The elimination of the GlyR-Abs with a gluten free diet suggests that, in the context of CD-related neurological dysfunction, these antibodies may be an epiphenomenon to the underlying gluten-driven pathophysiology.

Evidence of pathogenicity for these antibodies does exist. In vitro and in vivo studies have demonstrated that binding of GAD by anti-GAD antibodies suppresses GABA release with an epitope dependence, leading to the development of cerebellar ataxia, thus suggesting that GAD antibodies may be implicated in the pathophysiology of anti-GAD ataxia [15]. Similarly, GlyR-Abs pathogenicity on glycinergic neurotransmission has been recently demonstrated in vitro [16]. In this study purified IgG from patients with PERM or SPS who were positive for GlyR-Abs were observed to disrupt glycinergic neurotransmission.

A clinical spectrum of neurological disease associated with GlyR-Abs has been described [7]. Indeed at least 25% of patients with GlyR-Abs had other autoimmune disorders [6]. Immuno-modulatory therapy is often used in these patients. It is unclear if the adoption of gluten-free diet would aid recovery of the underlying autoimmune process in this patients’ population. Our 2 cases suggest that GlyR-Abs can be found in the context of CD-related brain hyperexcitability syndromes but are likely to be an epiphenomenon rather than being pathogenic. It is also possible that these antibodies are indeed pathogenic but the driver for GlyR autoimmunity is in fact the gluten sensitivity. This concept is not novel. Ventura et al. have made the observation that the prevalence of additional autoimmune diseases in children with CD is significantly lower than in those patients with CD diagnosed in adulthood [17]. They concluded that GFD may reduce the risk of developing additional autoimmune diseases later on in life. This observation echoes our previously observed reduction in anti-GAD antibodies in patients with anti-GAD related diseases and gluten sensitivity who go on a strict GFD [3].

Brain hyperexcitability can be a prominent feature in some patients with CD and neurological dysfunction [2]. This usually takes the form of cortical myoclonus and ataxia. These patients seem to have a propensity to refractory CD and rarely can progress to enteropathy associated T-cell lymphoma, as was the case in patient 1. A transcranial magnetic stimulation study demonstrated that de novo patients with CD have an imbalance in the excitability of cortical facilitatory and inhibitory circuits. Gluten-free diet was able to modulate the electrocortical changes in these CD patients. The length of dietary adherence to gluten-free diet was also an important factor in improving these electrocortical changes [18].

We conclude that a small number of patients with neurological manifestations of CD and in particular those with hyperexcitable brain syndromes may have GlyR-Abs. Gluten-free diet may be an effective therapeutic intervention in such patents. Such diet seems to also result in serological elimination of GlyR-Abs at least in one case reported here. Confirmation of this observation in a larger series would be desirable.

## Data Availability

Anonymised date can be available if requested.
